# Transforming Rural Health: States Should Invest Federal Funds to Enhance Primary Care

**DOI:** 10.1111/jrh.70147

**Published:** 2026-04-07

**Authors:** Johnny Huynh, Matthew Mackwood, Carrie Colla

**Affiliations:** ^1^ The Dartmouth Institute for Health Policy & Clinical Practice Geisel School of Medicine, Hanover New Hampshire USA; ^2^ Department of Community & Family Medicine Geisel School of Medicine, Hanover New Hampshire USA

For much of the 20th century, small towns and farming communities enjoyed health outcomes on par with those in cities. That parity vanished in the 1980s. Today, rural Americans carry a heavier burden of chronic disease and premature death, driven in part by higher rates of smoking and obesity [1]. Rural health systems are burdened with unstable financing and aging, underused infrastructure; clinician shortages and shrinking populations compound these problems [2, 3]. The recently enacted H.R. 1 directs $50 billion to the Rural Health Transformation Program (RHTP), presenting states with a rare opportunity to reimagine and rebuild the healthcare delivery systems their rural communities rely on.

RHTP funds come amid financial headwinds for rural providers. The $50 billion allocated through the program is a fraction of the Medicaid reductions that providers are projected to absorb beginning in 2027. In many states, these losses threaten to push already fragile clinics and hospitals into survival mode. As a result, states will understandably face pressure to use RHTP dollars to stabilize existing systems rather than to invest in long‐term transformation.

Primary care must anchor any serious rural health transformation. Robust primary care improves outcomes and lowers costs by providing continuous, comprehensive, and coordinated care [4, 5]. In rural communities, where specialty services are scarce or far away, primary care clinicians often serve as the first and only point of contact for care. Investing in payment reform, tele‐mentoring, and primary care infrastructure offers the clearest path to closing access gaps and building a sustainable rural health system.

No program better illustrates the promise and the limits of rural health transformation than the Rural Health Clinic (RHC) program. Established in 1977, it aimed to expand primary care access in rural and underserved communities by authorizing enhanced reimbursement to cover the higher costs inherent to rural care delivery infrastructure. Today, more than 5200 RHCs operate across the nation. Growth, however, has been uneven. Few practices sought RHC designation during the program's first decade, as our original analysis shows. Subsequent federal investments—the Omnibus Budget Reconciliation Act of 1989, the Medicare Modernization Act of 2003, and the Consolidated Appropriations Act of 2021—expanded the program by increasing reimbursement, broadening covered services, and raising payment caps. Yet, the program's long‐term stability remains uncertain. Many RHCs struggle to recruit and retain clinicians, integrate behavioral health, and modernize their infrastructure [6]. Operating on thin margins, most focus on survival rather than transformation.

This year, states have a rare opportunity to turn their obligation to rural communities into action. But transformation will only occur if policymakers resist the impulse to use RHTP funds as stopgap financing. Stabilization may be needed in the short term, but without parallel investments in delivery reform, rural systems will remain structurally vulnerable to future fiscal shocks. Investments should prioritize payment systems that sustain frontline providers while supporting the integration of behavioral health and chronic disease management. Regulators could, for instance, require health plans to allocate a greater share of spending to primary care and to strengthening interprofessional care teams. Empowering nurse practitioners, community health workers, pharmacists, and behavioral health providers would further extend the reach and resilience of rural primary care.

We see several promising areas for transformation to ensure that the long‐term health of rural populations keeps up with those of urban ones. The first is payment reform. Fee‐for‐service rewards volume over value and leaves little flexibility for innovation, especially in rural America where populations can be in decline. In contrast, global budgets and population‐based payment models better align incentives with prevention, health promotion, and community engagement. Pennsylvania's Rural Health Redesign Demonstration, for example, used prospective, population‐based payments to stabilize variable and declining funds for rural hospitals and redirect resources toward community health outside of traditional clinic visits. Although results have been mixed, the experience offers lessons on how to refine accountability structures, strengthen local partnerships, and balance financial stability with transformation goals.

Second, the Extension for Community Healthcare Outcomes (ECHO) model connects rural clinicians with specialists through structured tele‐mentoring. Originally developed to expand access to hepatitis C treatment, ECHO has since broadened to behavioral health, cancer care, and many other areas where specialty support is not close at hand. It has been associated with improved clinician expertise and patient outcomes, as well as reduced costs for management of expensive conditions like hepatitis C [7]. States could support new ECHO expansion sites, specialty tracks, or regionally tailored tele‐mentoring networks, reducing the need for long‐distance referrals and equipping rural practices to better manage chronic disease.

Finally, states can modernize the RHC program. Figure 1 shows that the number of RHCs surged after major federal investments and accelerated again after 2020. Growth has concentrated in the South and Midwest, where rural populations are largest. Rather than simply adding more clinics, states should update payment systems to incentivize care coordination, behavioral health integration, and health information technology. These updates would transform existing RHCs into hubs of comprehensive, community‐based care. Vermont's Blueprint for Health demonstrates how funding community health teams for coordination and behavioral health can guide RHC modernization in other states.

**FIGURE 1:**
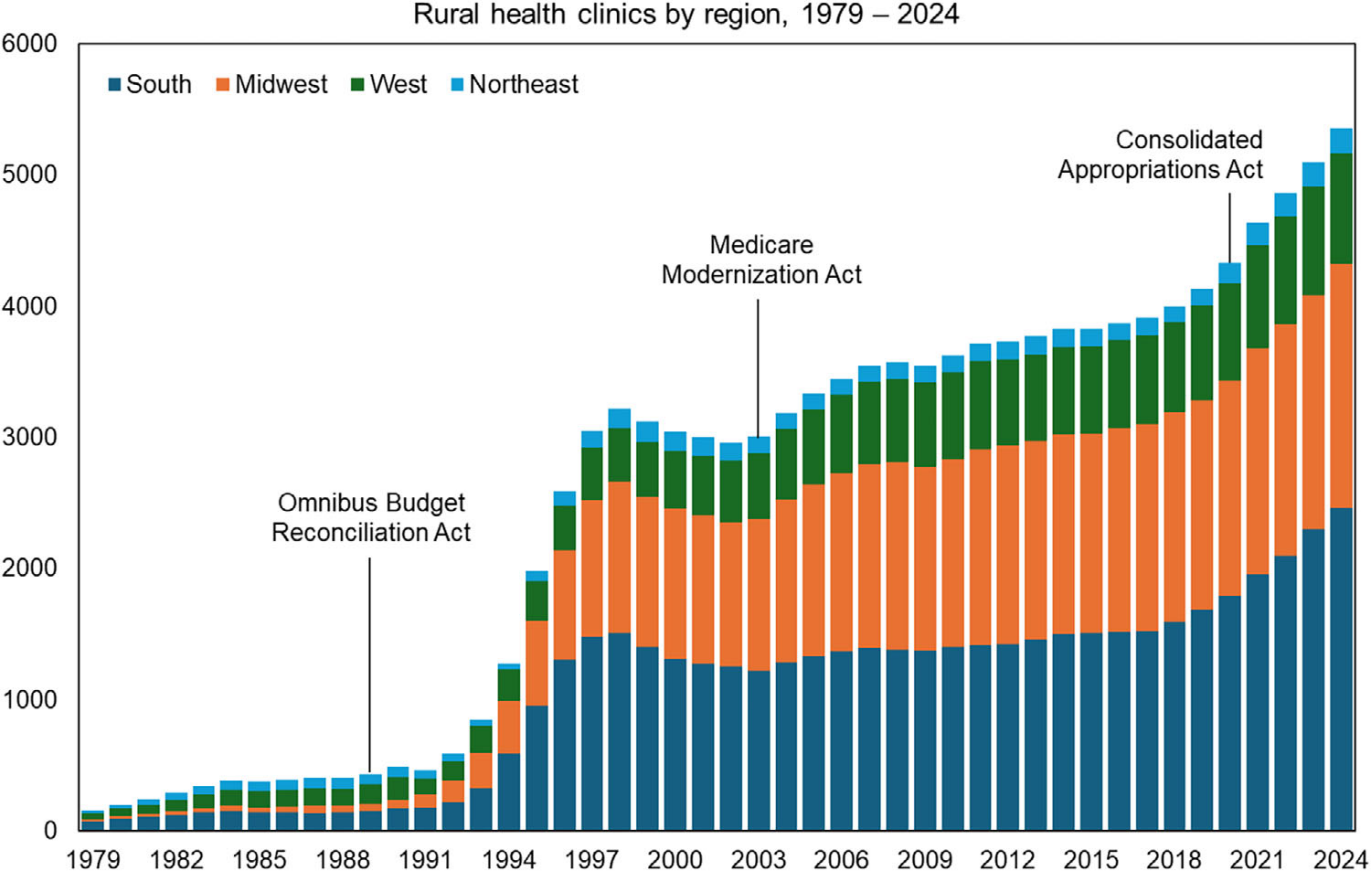
Number of Rural Health Clinics (RHCs) in the United States, 1979–2024, by Census region. Adoption of key policy milestones relevant to RHCs (the Omnibus Budget Reconciliation Act, Medicare Modernization Act, and Consolidated Appropriations Act) are annotated. Data are from the 2024 CMS Place of Service files.

RHTP will sunset after 5 years, leaving behind hundreds of varied efforts to redesign rural care, which deserve close study to assess their impact. Stakeholders must act now to plan rigorous evaluations: What worked, what failed, and why? Building strong research‐practice partnerships is key to that effort. Dartmouth Health, the nation's most rural academic medical center, illustrates this approach. Through initiatives like Northern New England Systems Transformation for Primary Care, researchers collaborate with rural hospitals, federally qualified health centers, and community practices to evaluate models grounded in the realities of rural delivery. Other institutions can advance similar work by forming data‐sharing partnerships across sectors and community organizations, embedding research within delivery systems, and engaging communities as equal partners in evaluation.

Those of us who live rurally know that life here involves tradeoffs—longer travel times, smaller facilities, and fewer specialists—but access to essential services should not be one of them. States should not treat RHTP as a temporary infusion; they must use it to build sustainable systems of care. By centering reform on primary care, expanding tele‐mentoring through ECHO, modernizing payment systems, and strengthening the RHC program, states can help reverse decades of decline in rural health and balance the independence of rural life with the security of reliable care.

## Conflicts of Interest

The authors declare no conflicts of interest.
